# Association between fatigue, peripheral serotonin, and L-carnitine in hypothyroidism and in chronic fatigue syndrome

**DOI:** 10.3389/fendo.2024.1358404

**Published:** 2024-03-05

**Authors:** Tommi Raij, Kari Raij

**Affiliations:** ^1^ Department of Physical Medicine and Rehabilitation, Feinberg School of Medicine, Northwestern University, Chicago, IL, United States; ^2^ Department Of Neurobiology, Weinberg College of Arts and Sciences, Northwestern University, Chicago, IL, United States; ^3^ MGH/MIT/HMS Athinoula A. Martinos Center for Biomedical Imaging, MGH Department of Radiology, Boston, MA, United States; ^4^ Kruunuhaka Medical Center, Helsinki, Finland

**Keywords:** peripheral serotonin, L-carnitine, fatigue, hypothyroidism, chronic fatigue syndrome (CFS), myalgic encephalomyelitis (ME), systemic exertion intolerance disease (SEID), mitochondria

## Abstract

**Background:**

Fatigue of unknown origin is a hallmark symptom in chronic fatigue syndrome (CFS) and is also found in 20% of hypothyroidism patients despite appropriate levothyroxine treatment. Here, we suggest that in these disorders, peripheral serotonin levels are low, and elevating them to normal range with L-carnitine is accompanied with reduced fatigue.

**Methods:**

We conducted a retrospective analysis of follow-up clinical data (CFS N=12; hypothyroidism with fatigue N=40) where serum serotonin and fatigue levels were compared before *vs.* after 7 weeks of oral L-carnitine supplementation.

**Results:**

After L-carnitine, serotonin increased (8-fold in CFS, Sig. = 0.002, 6-fold in hypothyroidism, Sig. < 0.001) whereas fatigue decreased (2-fold in both CFS and hypothyroidism, Sig. = 0.002 for CFS, Sig. < 0.001 for hypothyroidism). There was a negative correlation between serotonin level and fatigue (for CFS, rho = -0.49 before and -0.67 after L-carnitine; for hypothyroidism, rho = -0.24 before and -0.83 after L-carnitine).

**Conclusions:**

These findings suggest a new link between low peripheral serotonin, L-carnitine, and fatigue.

## 1 Introduction

Fatigue is a common chronic cross-diagnostic symptom (1–4). Recently, some emerging evidence has suggested a possible association between low peripheral serotonin and fatigue. On the one hand, low peripheral serotonin has been found during post-infectious fatigue following COVID-19 and other viruses (5) and in fibromyalgia patients (6, 7). On the other hand, peripheral serotonin, once believed to mainly regulate intestinal mobility, is now understood to play key roles in regulating peripheral energy metabolism (8–12). Here, we examine if peripheral serotonin levels might be associated with fatigue in two other poorly understood conditions, chronic fatigue syndrome (CFS) and hypothyroidism with persistent fatigue.

In CFS, fatigue of unknown origin is a hallmark diagnostic feature (13). Its current prevalence in the U.S is 1.3% (14). Little is known about potential causes of CFS, though several recent studies point to activation of inflammatory cascades and issues with mitochondrial energy production (15–20). Yet, currently there is no established laboratory test that could be used to diagnose or monitor disease progression or severity. Metabolomic, cytokine, mitochondrial, and natural killer cell meta-analyses have revealed an abnormal but mixed pattern, likely originating from both heterogeneity across patients and study methods (21–24). Moreover, treatment is difficult and often leads to only partial alleviation of symptoms (13).

In hypothyroidism, about 15 – 20% of patients report fatigue or “brain fog” even after correction of thyroxine levels to normal with levothyroxine (25–28). Self-reported quality-of-life (QoL) measures also highlight fatigue as a key problem in hypothyroidism (29). However, the mechanism remains unknown.

L-carnitine supplementation has been suggested to reduce fatigue in both hypothyroidism (26) and in CFS (30, 31). Indicating a putative link between carnitine and serotonin, studies in mice have shown that carnitine supplementation increases serotonin concentration in the brain (32, 33). Given that serotonin synthesis pathways are similar between the brain and periphery and across mammalian species, we here examine in humans the following hypotheses in both CFS and hypothyroidism with fatigue: (1) serum serotonin (S-5-HT) levels are low, (2) L-carnitine supplementation is associated with increased S-5-HT levels and reduced fatigue, and (3) there exists a correlation between peripheral S-5-HT and fatigue levels.

## 2 Materials and methods

### 2.1 Study design and participants

The present study is a retrospective analysis of follow-up clinical data. The design is non-randomized, and each patient served as their own control (i.e., each patient was tested before and after L-carnitine supplementation). Ethical approval was obtained from the IRB committees at Faculty of Medicine, University of Helsinki, Helsinki, Finland, and at Feinberg School of Medicine, Northwestern University, Chicago, Illinois, USA. The sample consists of consecutive patients seen by the senior author over a 15-month period for CFS (5/2/2016 – 8/23/2017) and an 11-month period for hypothyroidism (11/1/2016 – 10/9/2017). Patients had been previously diagnosed with autoimmune hypothyroidism (initial N = 312) or CFS (initial N = 16). In addition, to be included in the sample, it was required that each patient spontaneously reported fatigue and chose to receive oral L-carnitine supplementation for 7 weeks, resulting in N = 52 for hypothyroidism (17% of patients) and N = 16 for CFS (all patients). Our L-carnitine dose was 300 mg twice a day, which falls within the typical range of 250 – 3000 mg total dose per day across and within studies [*e.g.*, (34)]. The duration of 7 weeks was chosen based on a prior human study in CFS patients showing that the greatest improvement against fatigue took place between 4 and 8 weeks (30) and rodent studies showing effects on brain serotonin levels already after 21 – 25 days of carnitine supplementation (32, 33). Patients that had received selective serotonin reuptake inhibitors (SSRIs) within the past 6 months were excluded, since SSRIs may alter S-5-HT levels (35), resulting in exclusion of 7 hypothyroidism and 2 CFS patients. Patients that did not return signed consent forms (N = 5 for hypothyroidism, N = 1 for CFS) were excluded. Finally, 1 CFS patient was removed during data analysis due to receiving a diagnosis of autoimmune thyroiditis after the study time window. All patients that satisfied these criteria were included, resulting in a final sample of 40 hypothyroidism (age mean 46 years, range 20 – 79 years, 4 males) and 12 CFS (mean age 43 years, range 23 – 68 years, 3 males) patients. **Table 1** shows the detailed inclusion, exclusion, and removal criteria. In the sample with autoimmune hypothyroidism, the diagnosis had been made by endocrinologists at the Helsinki University Central Hospital, where the diagnostic criteria included elevated TPO antibodies, elevated TSH, reduced FT4, and ultrasound findings characteristic of autoimmune thyroiditis. All patients were on stable levothyroxine treatment and were, according to clinical and laboratory results, currently euthyreotic (**Table 2**). In CFS, the diagnosis had been made by internal medicine specialists at the Helsinki of University Central Hospital, where the diagnostic criteria followed the 2003 Canadian Consensus Criteria (36). All CFS patients had been found to be euthyreotic during the initial diagnosis as well as shortly before L-carnitine (**Table 2**, samples taken 1 – 4 months before starting L-carnitine administration).

**Table 1:** Inclusion, Exclusion, and Removal Criteria.

Inclusion criteria for hypothyroidism with fatigue	
- Clinical diagnosis of hypothyroidism > 3 years earlier - During initial diagnosis, laboratory tests indicated autoimmune origin (elevated TPO-AB) - Steady levothyroxine dose for at least 2 years - TSH and T4F levels within normal range before L-carnitine - Spontaneous report of fatigue during a control visit
Inclusion criteria for CFS	
- Clinical diagnosis of CFS (with symptoms lasting >6 months) - Spontaneous report of fatigue during a control visit
Exclusion criteria for both groups	
- Age below 20 years - Known untreated disease where diagnostic criteria include fatigue - Thyroid medications other than levothyroxine (triiodothyronine) within the past 6 months - SSRI medication within the past 6 months - Pregnancy
Removal criteria for both groups	
- Receiving diagnoses for both CFS and hypothyroidism

**Table 2:** Subject Demographics and Clinical/Laboratory Test Results (mean ± SD).

Subject demographics and clinical/laboratory test results (other than S-5-HT)				
	HT+Fatigue	CFS	Ref range	Unit
Age	47 ± 16	43 ± 16	—	Years
Sex	36 F/4 M	9 F/3 M	—	
Race (all)	Caucasian	Caucasian	—	
Ethnicity (all)	Not Hispanic or Latino	Not Hispanic or Latino	—	
BMI	26 ± 4	27 ± 5	< 25	kg/m^2^
Heart rate	68 ± 5	71 ± 5	60 – 100	bpm
SpO2	97 ± 1	97 ± 2	> 95	%
B-Hb	128 ± 9	133 ± 12	117 – 155 F 134 – 167 M	g/l
B-HbA1C	31 ± 5	33 ± 6	20 – 42	mmol/mol
P-CRP	< 3 for each	< 3 for each	< 3	mg/l
P-TSH	1.6 ± 0.7	1.5 ± 0.8	0.5 – 3.6	mU/l
P-FT4	14.8 ± 2.3	13.8 ± 2.4	9 – 19	pmol/l
P-Hcyst	7.2 ± 1.3	8.1 ± 2.1	5.0 – 15.0	µmol/l

These tests were taken only once, before starting carnitine supplementation. All values except BMI were within normal range. HT+Fatigue, Hypothyroidism+Fatigue; CFS, Chronic Fatigue Syndrome; BMI, Body-Mass Index; B-Hb, Blood Hemoglobin; B-HbA1C, Blood Glycated Hemoglobin; P-CRP, Plasma C-Reactive Protein; P-TSH, Plasma Thyroid-Stimulating Hormone; P-FT4, Plasma Free T4; P-Hcyst, Plasma Homocysteine.

### 2.2 Outcomes and other collected variables

S-5-HT levels and behavioral fatigue levels had each been recorded twice, once before and once after the L-carnitine challenge. Specifically, on the 1^st^ physician visit (Week 0), the first fatigue score was recorded. The 1^st^ laboratory S-5-HT test was one week later (Week 1) followed by starting the L-carnitine regimen. The 2^nd^ S-5-HT laboratory test was six weeks later (Week 7), and the 2^nd^ physician visit with the second fatigue score one week thereafter (Week 8). On each physician visit, the fatigue score had been measured before discussing laboratory test results. Details of each are described below.

### 2.3 Fatigue score measurement

During the clinical visits, the treating physician recorded the fatigue level using Question 1 adapted from the Bristol rheumatoid arthritis fatigue numerical rating scale (BRAF-NRS) (37). Specifically, the patients were asked “How fatigued have you been on average during the past week on a scale of 0 – 10, zero meaning as badly fatigued as you can imagine and 10 meaning not fatigued at all” (translated from Finnish). During data analysis, to be consistent with the convention where larger values reflect increasing symptom severity, the raw score axis was flipped (*i.e.*, the raw values were subtracted from 10), resulting in final fatigue scores where 0 = no fatigue and 10 = maximal fatigue. Note that L-carnitine supplementation, started on Week 1 after the 1^st^ S-5-HT laboratory visit, continued until the 2^nd^ physician visit on Week 8 when the second fatigue score value was recorded.

### 2.4 S-5-HT assay

The S-5-HT (as well as all other laboratory) samples were collected at the Helsinki University Hospital Laboratory, where the S-5-HT protocol includes dietary restrictions for two days prior to the sample and collecting the samples between 8 – 10 am. The dietary instructions were given in writing and read aloud to the patients by the treating physician (“For 2 days before the laboratory visit, do not consume foods containing serotonin or tryptophan, including avocado, banana, plum, peanuts, hickory nuts, pineapple, eggplant, tomato, kiwi, grapefruit, and honeydew”, translated from Finnish). There was no fasting requirement. The laboratory personnel verbally confirmed that the patient had been aware of the dietary restrictions before each blood draw. S-5-HT was measured with the HPLC-ECD chromatographic method (38) using a fully clotted sample to accurately take into account the serotonin in circulating platelets. Specifically, blood was drawn into a pre-cooled serum test tube wrapped in a cold gel bag, mixed, and allowed to clot in cold at least 60 minutes. Then, the sample was centrifuged in cold, after which the serum was extracted and frozen. Next, the serum sample was shipped frozen overnight to a DAkkS accredited laboratory (L.A.D.R. GmbH, Labor Dr. Kramen & Kollegen, Geesthact, Germany) where the S-5-HT assays were conducted every 5 days and the samples were kept frozen until analysis. In this laboratory, intra-assay variation of the method has been reported at 4.5% (CV%) and the inter-assay variation at 4.7% (CV%). The detection limit was 15.9 nmol/l (2.8 µg/l) and the assay was linear between 45 and 8041 nmol/l (8 and 1417 µg/l). The 1^st^ and the 2^nd^ samples were analyzed independently, as soon as possible after each blood draw, using the same method described above.

### 2.5 Other data

In addition, the following clinical routine values had been measured once before the L-carnitine regimen: thyroid function (TSH, FT4), Hb, HbA1C, CRP, homocysteine, BMI, pulse rate, and oxygen saturation. All but BMI were within normal range (**Table 2**), which excludes frequent conditions that may independently cause fatigue (insufficient levothyroxine dose, anemia, diabetes with poor glycemic control, infection). All patients were non-smokers (39). Other diagnoses and medications were documented.

### 2.6 Statistical analysis

Some of the S-5-HT and fatigue scores were not normally distributed (Shapiro-Wilk test). Therefore, changes in fatigue and S-5-HT levels were examined with Related-Samples Wilcoxon Signed Rank Test within-subjects (before vs. after L-carnitine challenge). Correlations (simple and partial) between fatigue and S-5-HT levels were assessed using Spearman *rho* correlation coefficients. In addition, we conducted two *post-hoc* partial correlation analyses to examine possible confounding effects of age or TSH level. Finally, we computed subgroup control analyses based on the absence or presence of co-occurring diagnoses (Related-Samples Wilcoxon Signed Rank Test within-subjects before vs. after L-carnitine challenge, Spearman *rho* simple correlations between fatigue and S-5-HT levels). All significance (Sig.) values were 2-tailed. All statistics were computed using SPSS (version 28; IBM, Chicago, IL, USA).

## 3 Results

### 3.1 Patient samples

The hypothyroidism sample (N = 40) included the following co-occurring diagnoses and conditions (N = 21) made at least 1 year before starting L-carnitine supplementation in the study: hypertension (N = 6), type 2 diabetes (N = 6), sleep apnea managed with CPAP treatment (N = 3), Sjögren’s Syndrome (N = 2), vitiligo (N = 2), hypercholesteremia (N = 2), menopausal symptoms (N = 2), asthma (N = 1), celiac sprue (N =1), and Meniere’s Disease (N = 1). There were no new diagnoses within 1 year or during the L-carnitine supplementation. Medications were unchanged for at least 4 months before and during the study. While most of the medications should not have influenced the S-5-HT level, one participant was taking Coenzyme Q_10_ (CoQ_10_) 100 mg once a day.

The CFS sample (N = 12) included to following co-occurring diagnoses (N = 6) made at least 1 year before starting L-carnitine supplementation in the study: asthma (N =1), allergic rhinitis (N = 1), celiac sprue (N = 1), type 1 diabetes (N = 1), prostate hypertrophy (N = 1), and gastro-esophageal reflux disease (N = 1). Again, there were no new diagnoses within 1 year or during the L-carnitine supplementation, and medications were unchanged for at least 4 months before and during the study.

### 3.2 S-5-HT levels and their changes

**Figure 1:**
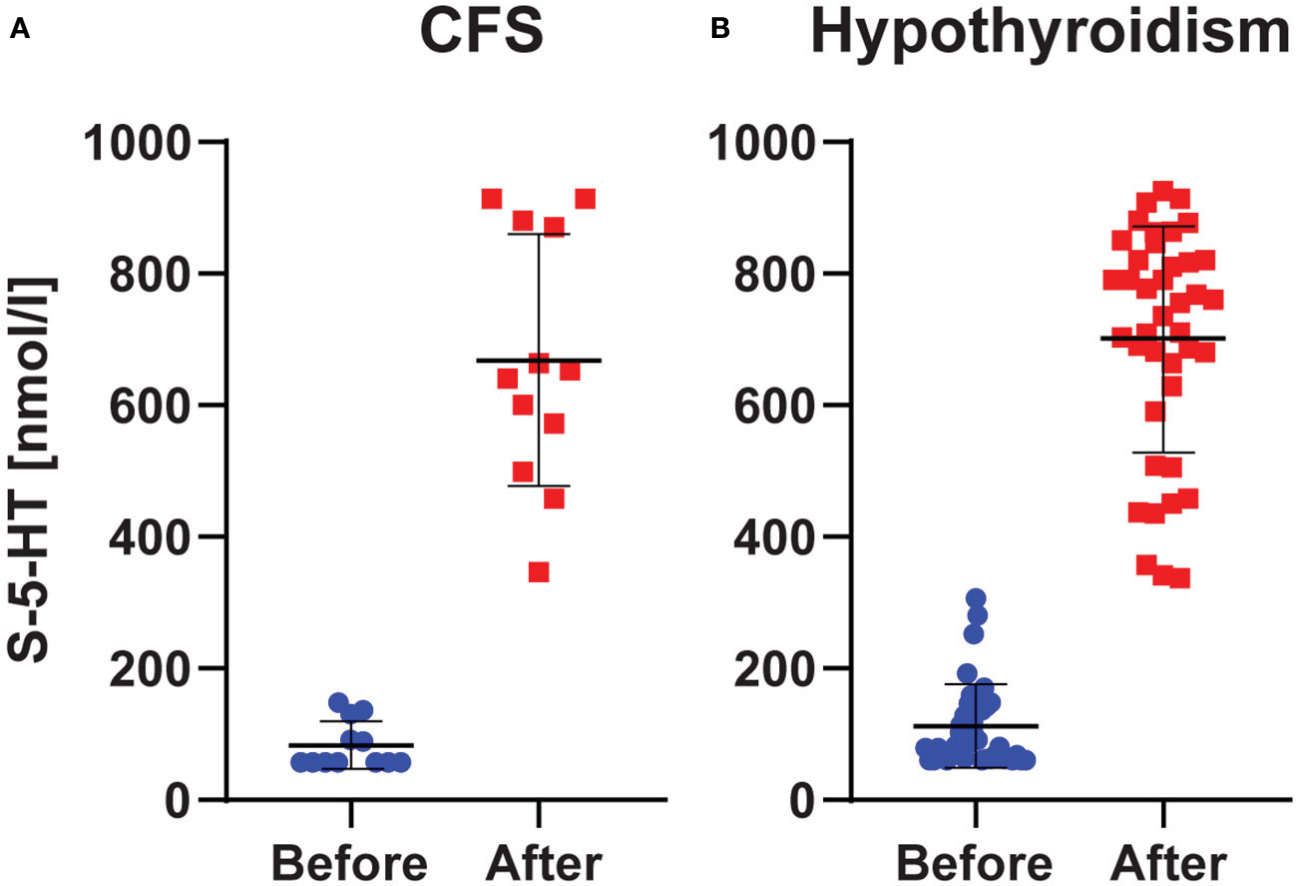
S-5-HT Levels. Levels in chronic fatigue syndrome **(A)** and hypothyroidism with fatigue **(B)** before and after L-carnitine. Blue and red dots represent the individual observations, whereas the horizontal black lines show the mean ± SD values.

The normal range for the employed S-5-HT test was 350 – 825 nmol/l. Below, across-subjects results are reported as (mean (median) ± SD). Before L-carnitine, S-5-HT was markedly low in both CFS (83 (57) ± 36 nmol/l), and hypothyroidism (112 (84) ± 63 nmol/l). After L-carnitine, S-5-HT levels were elevated to normal range in both CFS (668 (647) ± 190 nmol/l) and hypothyroidism (698 (748) ± 170 nmol/l). Thus, this increase in S-5-HT from before to after L-carnitine was about 8-fold in CFS and 6-fold in hypothyroidism. This increase was statistically significant in both groups (Sig. = 0.002 for CFS and Sig. < 0.001 for hypothyroidism) (**Figure 1**).

### 3.3 Fatigue score changes

**Figure 2:**
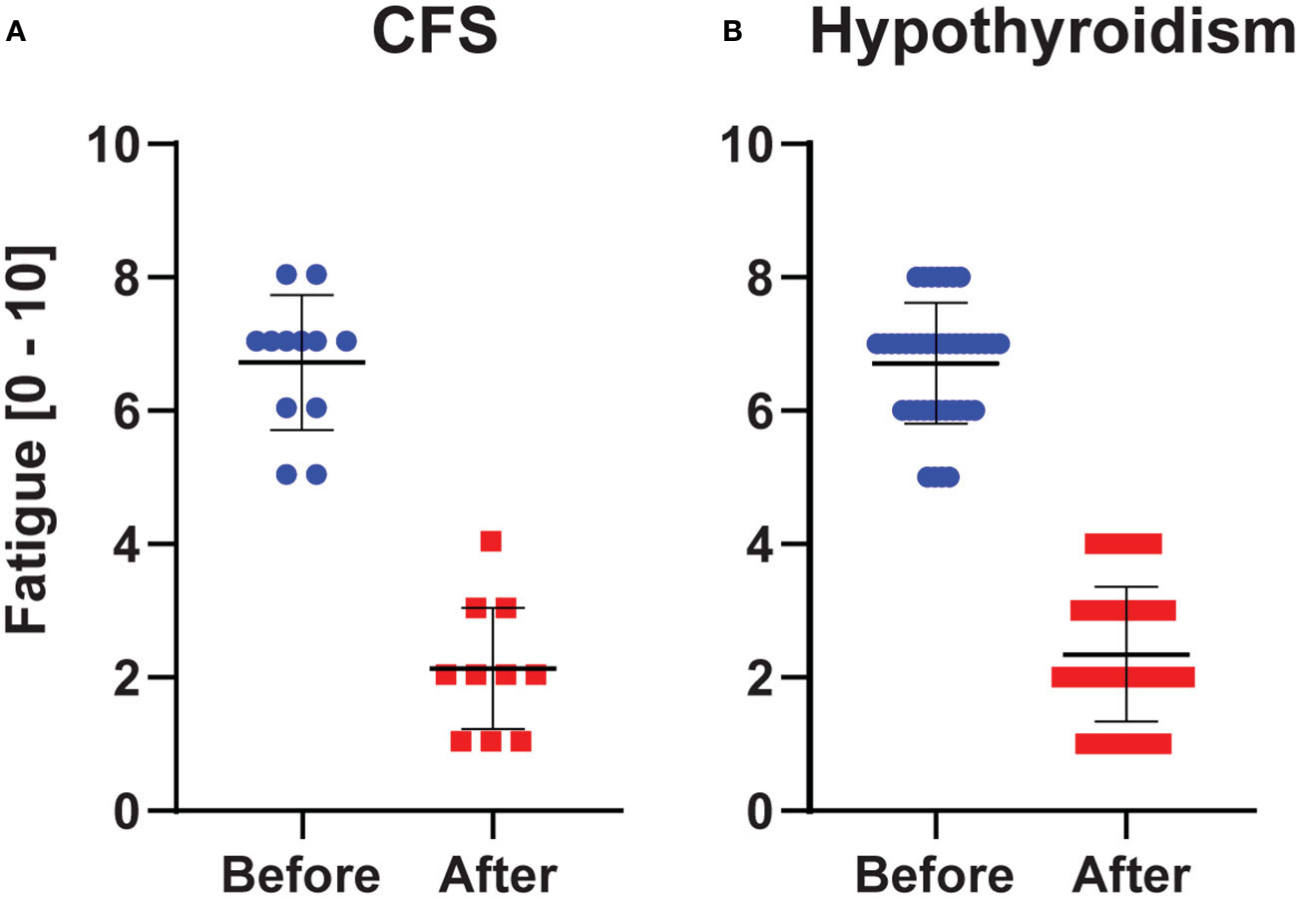
Fatigue Scores (0 = no fatigue, 10 = worst imaginable fatigue). Values in chronic fatigue syndrome **(A)** and hypothyroidism with fatigue **(B)** before and after L-carnitine. Blue and red dots indicate the individual observations, whereas the horizontal black lines show the mean ± SD values.

The fatigue scores decreased about 2-fold from before to after L-carnitine supplementation in both hypothyroidism (from 6.7 (7.0) ± 0.9 to 2.4 (2.0) ± 1.0; Sig. < 0.001) and CFS (from 6.7 (7.0) ± 1.0 to 2.1 (2.0) ± 0.9; Sig. = 0.002) (**Figure 2**).

### 3.4 Correlations between S-5-HT and fatigue levels

**Figure 3:**
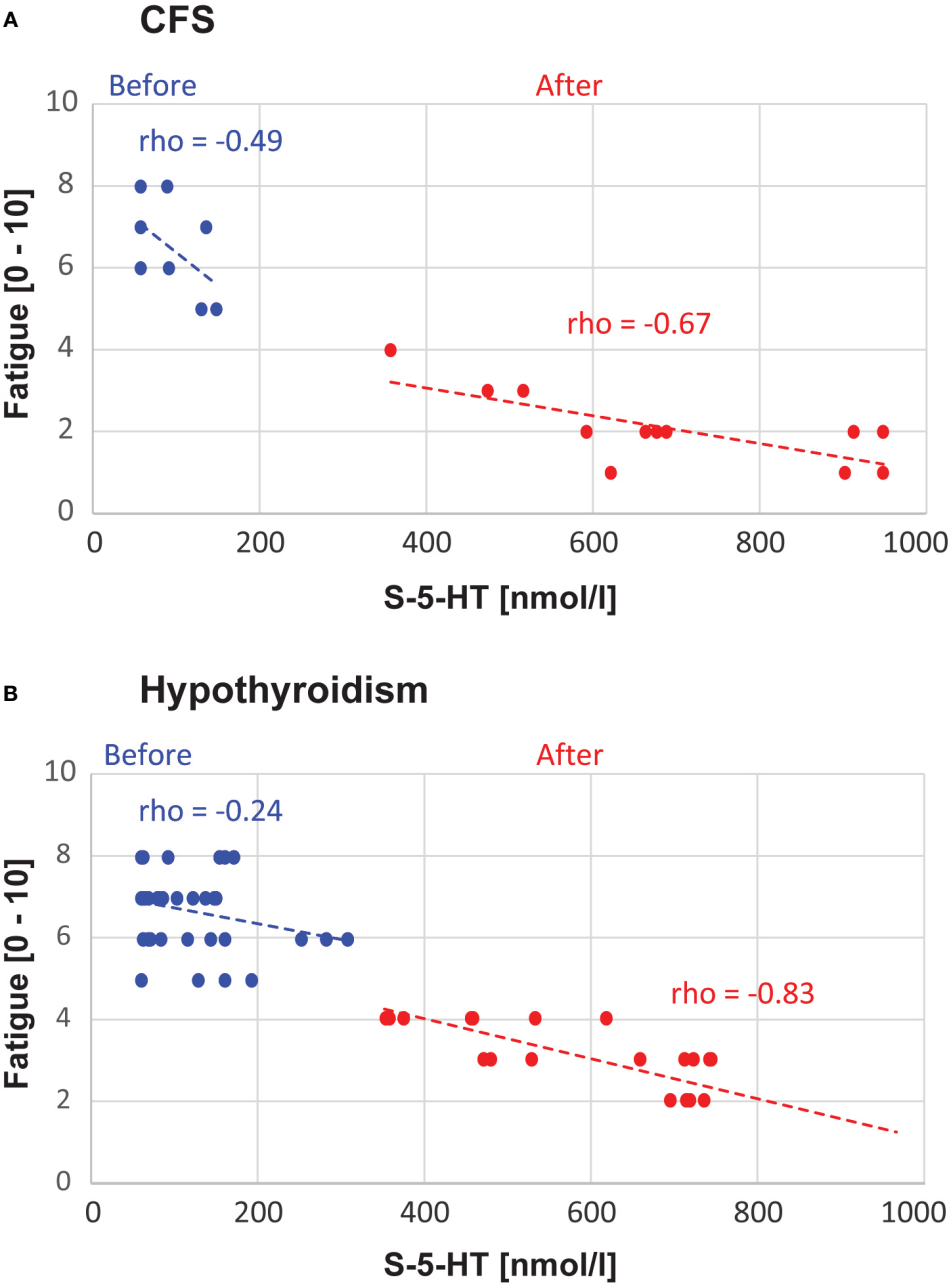
Correlation Analyses. The panels show the data points and correlations between S-5-HT and fatigue level, separately for chronic fatigue syndrome (CFS, **A**) and hypothyroidism with fatigue **(B)**. The Spearman *rho* values and linear trendlines are shown separately for before *vs.* after L-carnitine.

There was a negative correlation between S-5-HT and fatigue scores, such that increased serotonin levels were associated with reduced fatigue. Specifically, in CFS, the correlation showed a non-significant trend before (*rho* = -0.49 Sig. = 0.109), and a significant correlation after (*rho* = -0.67 Sig. = 0.016) L-carnitine supplementation. Similarly, in hypothyroidism, before carnitine the correlation showed a non-significant trend (*rho* = -0.24 Sig. = 0.131), whereas after L-carnitine the correlation was significant (*rho* = 0.83 Sig. < 0.001) (**Figure 3**).

### 3.5 Control analyses for age or TSH level as confounding factors

In addition to the above planned statistical tests, we conducted two *post hoc* partial correlation analyses to examine possible confounding effects of age or TSH level as confounding factors. With age as a covariate, the Spearman *rho* values remained practically unchanged (differed by less than 0.02) regardless of if we excluded or included the covariate, separately for all 4 conditions [Hypothyroidism/CFS x Before/After L-carnitine]. With the TSH level (available only before L-carnitine, since these were clinical data) as a covariate, in CFS the results again remained similar (*rho* value differences < 0.07). However, in hypothyroidism before L-carnitine, there was a correlation (*rho* = 0.376, Sig. = 0.017) between fatigue scores and TSH, which led to underestimation of the simple correlation between S-5-HT and fatigue scores (without TSH as a covariate, *rho* = -0.24 Sig. = 0.131 as reported above, with TSH as a covariate *rho* = -0.364 Sig. = 0.023). After L-carnitine, this confounding effect of TSH disappeared (*rho* value difference < 0.01).

### 3.6 Control analyses for co-occurring diagnoses

Finally, to examine if the absence *vs.* presence of any co-occurring diagnoses influenced the results, in another *post hoc* analysis we split each group into two subgroups: those that had no other diagnosis, and those that did have one or more additional diagnoses (for hypothyroidism, N = 19 and N = 21; for CFS, N = 6 and N = 6, respectively). Independently in each of the 4 subgroups, the changes in both S-5-HT and fatigue level from before to after L-carnitine remained significant (Sig. < 0.001 for each hypothyroidism subgroup; Sig. = from 0.026 to 0.028 for each CFS subgroup). The correlations between S-5-HT and fatigue were also similar. Specifically, for hypothyroidism, all correlations that were significant or non-significant remained so in the subgroup analyses. For CFS, the subgroup without co-occurring diagnoses showed the same pattern of non-significant correlation before and significant correlation after L-carnitine; in contrast, the subgroup with co-occurring diagnoses flipped into significant correlation before and non-significant correlation after L-carnitine, most likely reflecting that at N = 6 it becomes difficult to obtain reliable correlation estimates. Overall, the subgroup analyses indicated that co-occurring diagnoses did not influence the main results or conclusions.

## 4 Discussion

The serum serotonin levels were low before L-carnitine, L-carnitine supplementation was associated with a robust increase in peripheral serotonin and reduction of fatigue, and there was a statistically significant correlation between serum serotonin and fatigue levels in both patient groups. Thus, the results supported our hypotheses. This finding is mechanistically interesting because there are no published direct links between carnitine and peripheral serotonin synthesis. Further, to our knowledge, within-subjects correlations between S-5-HT and fatigue levels have not been previously reported in humans in any disorder. The fact that the findings were similar across CFS and hypothyroidism points to the possibility that low peripheral serotonin may be a common theme across some disorders that include fatigue. The control analyses suggested that age or co-occurring diagnoses did not bias the results. These findings and interpretations are indirectly supported by a recent report on fatigue in the long COVID-19 syndrome where lower peripheral serotonin was observed in patients than in healthy controls (5). To arrive at putative mechanisms, below we discuss 5-HT and carnitine, along with their possible links to fatigue.

Peripheral 5-HT is produced and secreted by the enterochromaffin cells in the gastrointestinal tract where it regulates intestinal mobility. Much of the peripheral 5-HT is picked up from the intestine by, and circulating in, platelets. In addition to intestinal mobility, it has become evident that peripheral serotonin participates in systemic energy homeostasis (10, 12). S-5-HT is produced in a two-step enzyme reaction from the essential amino acid tryptophan. The first reaction is catalyzed by tryptophan hydroxylase (TPH-1), which requires L-tryptophan, tetrahydrobiopterin (BH4), and O_2_ as co-substrates, and has ferrous ion (Fe^2+^) as an activator. The second enzyme, L-aromatic amino acid decarboxylase (AADC a.k.a. AAAD), changes the intermediate product to 5-HT. This step has 5-hydroxy-L-tryptophan as substrate, pyridoxal 5’-phosphate as an optional activator, and is influenced by Al^3+^, Zn^2+^, Ca^2+^, and Mg^2+^ levels.

Peripheral serotonin in hypothyroidism and CFS has previously received little attention, and S-5-HT measurements have been used mainly in the carcinoid tumors/syndrome. Current serotonin medications are aimed at increasing central 5-HT neurotransmission in the brain, and there are no approved indications for manipulating peripheral 5-HT, though peripheral 5-HT synthesis is being considered as a potential target for developing pharmacological agents in some disorders (8, 9). Moreover, the peripheral and brain pools of 5-HT are synthesized separately and isolated from each other by the blood-brain-barrier; only the precursor 5-HTP passes the blood-brain barrier.

We are not aware of prior studies reporting low S-5-HT in hypothyroidism or CFS. A recent study suggested that plasma levels of 5-MT, a compound related to serotonin metabolism, may be decreased in CFS patients, but this has been interpreted as a confounder caused by serotonin-selective reuptake inhibitor (SSRI) use in these patients (40). The present results support the idea that peripheral serotonin metabolism may be altered in hypothyroidism and CFS. Theoretically, a low S-5-HT value would be expected to result from lack of its precursor tryptophan or disturbance in the two-step synthesis described above. Further, while low tryptophan dietary intake is rare, CFS and autoimmune hypothyroidism may be associated with an increment in indeloamine-dioxygenase (IDO) expression, which would promote the tryptophan-kynurenine pathway that decreases serotonin synthesis. Specifically, when IDO activity increases, more tryptophan is diverted away from serotonin synthesis and toward kynurenine. Interestingly, the aryl hydrocarbon receptor (AhR) of the tryptophan-kynurenine pathway serves as a critically important mediator between tryptophan metabolism, immune reactions, and tissue homeostasis (41–43), and several kynurenine metabolites have been associated with fatigue (44, 45).

The principal role of carnitine is to transport long-chain fatty acids into the mitochondria for energy production via beta-oxidation (46), but it also participates in glucose metabolism, detoxification of acyl moieties (47), and in the preservation of intracellular Coenzyme A (CoA) homeostasis (48). In some open-label trials, carnitine supplementation has been suggested to reduce CFS symptoms (30, 31). However, studies examining carnitine levels in CFS patients have been inconclusive, showing either normal (49, 50) or reduced levels (30, 40, 51, 52) for at least some acylcarnitine forms (53).

While carnitine supplementation could have benefits, some recent studies suggest that chronic oral L-carnitine administration may be associated with an increased risk for atherosclerosis via increased trimethylamine (TMA) and/or trimethylamine N-Oxide (TMAO) levels (54, 55). If true, this would not favor long-term L-carnitine supplementation, or may require the development of additional strategies to reduce TMA/TMAO levels (56). Yet, at this point, it is unclear if L-carnitine promotes atherosclerosis (57) and the relationship seems to be context dependent (58).

Mitochondrial dysfunction has been suggested to contribute to CFS (15–18) and symptom severity seems to correlate with the degree of mitochondrial dysfunction (19) [for reviews, see (59–62)]. Since carnitine enhances mitochondrial function, it is plausible that the reduced fatigue levels observed in the present study were mediated by carnitine-driven improvement in mitochondrial function. It is possible that functional deficits in the mitochondria of CFS patients require higher than normal carnitine levels to support sufficient energy production. Alternatively, if the functional deficit in CFS patients leads to recruitment of abnormal ATP production pathways (63), carnitine supplementation might restore the use of normal reaction pathways, thus promoting metabolic flexibility.

The mechanisms that link carnitine intake, S-5-HT, and fatigue, in both CFS and hypothyroidism, are yet unknown. However, in the mouse brain, L-carnitine supplementation has been shown to increase brain 5-HT concentration (32, 33). Perhaps most tellingly, carnitine and 5-HT share the same cellular target organ, since carnitine participates in energy production at the mitochondrial level, and 5-HT receptors have a role in mitochondrial function in muscle tissue (11). As a putative bridge between carnitine and S-5-HT, increased energy production allowed by L-carnitine supplementation might promote peripheral 5-HT synthesis. This idea is indirectly supported by a study in fibromyalgia patients where supplementation with another key mitochondrial agent, CoQ_10_, increased low peripheral serotonin values (7).

Limitations of the study include a relatively small non-randomized retrospective clinical sample. As typical for retrospective chart reviews, the outcome variables were limited to those that were systemically recorded on the patient charts during routine clinical outpatient visits, resulting in that some tests (*e.g.*, corticotropin-releasing hormone stimulation test) that may have been helpful will need to be conducted in future prospective trials. Further, since this was an investigation focused *a priori* on S-5-HT levels, it remains unknown if the effects were specific to serotonin synthesis, or if other pathways were also affected. Moreover, it is not known if S-5-HT mediated the effects of L-carnitine on fatigue, as it is possible that L-carnitine could have effects unrelated to S-5-HT that could reduce fatigue and in parallel promote increased synthesis of peripheral 5-HT. Finally, for recording fatigue levels, the present study adopted BRAF-NRS Question 1 (37), which consists of a single item and is therefore well suited for clinical routine. Future prospective studies should use more extensive fatigue questionnaires, such as the Fatigue Assessment Scale (FAS) (64) or the 11-point fatigue numeric rating scale (NRS) (65, 66). We also note that, while NRS, BRAF-NRS, and FAS have been validated in some disorders, currently there is no single fatigue behavioral instrument that has been validated in both hypothyroidism and CFS.

Future directions include prospective randomized controlled trials where the present findings are replicated with more detailed questionnaires and other tests for fatigue, depression, sleep, and stress. It would also be pertinent to add laboratory assays that allow putting the results in the larger context of cellular senescence, which seems to be disturbed in several disorders where fatigue has been associated with mitochondrial dysfunction. Specifically, recent research has suggested a bidirectional communication between metabolic dysfunction (loss of metabolic homeostasis) and cellular senescence (67, 68). This relationship between metabolism and senescence has been considered for many chronic phenotypes, including for fatigue (69–75). Mitochondrial dysfunction has been implicated in the altered metabolic flexibility in such conditions (76, 77).

In conclusion, the present results suggest new and unexpected links between fatigue, peripheral 5-HT, and L-carnitine in CFS and hypothyroidism, and offer testable hypotheses for future mechanistic and clinical studies. Peripheral serotonin and its putative link with L-carnitine appear to emerge as new candidates for understanding and treating fatigue across several disorders.

## Data availability statement

The raw data supporting the conclusions of this article will be made available by the authors, without undue reservation.

## Ethics statement

The studies involving humans were approved by the University of Helsinki, Faculty of Medicine. The studies were conducted in accordance with the local legislation and institutional requirements. The human samples used in this study were acquired from a by-product of routine care or industry. Written informed consent for participation was obtained from the participants in accordance with the national legislation and institutional requirements.

## Author contributions

TR: Conceptualization, Formal analysis, Methodology, Supervision, Visualization, Writing – original draft, Writing – review & editing. KR: Conceptualization, Data curation, Formal analysis, Investigation, Methodology, Resources, Supervision, Writing – original draft, Writing – review & editing.
